# Doctoring Undercover: updating the educational tradition of shadowing

**DOI:** 10.1080/10872981.2017.1265848

**Published:** 2016-12-16

**Authors:** Claire D. Clark

**Affiliations:** ^a^ The Department of Behavioral Science, University of Kentucky College of Medicine, Lexington, KY, USA

**Keywords:** Premedical, interprofessional, interdisciplinary, behavioral and social sciences, active learning, reflective writing, online resource, pilot

## Abstract

**Background:** Premedical students are educated in basic biological and health sciences. As a complement to traditional premedical coursework, medical school applicants are encouraged to shadow practitioners, with the hope that observation will introduce students to the culture and practice of healthcare. Yet the shadowing experience varies widely across practitioners and institutions; resources that guide students’ critical reflection and structure the experience are scarce.

**Development:** A pilot experiential learning course, Doctoring Undercover: Shadowing and the Culture of Medicine, was developed to fill this gap. The course consisted of three parts: an introduction to medical culture through the disciplines of medical sociology, history, anthropology, and bioethics; a site placement in which students applied these fields’ analytical techniques to the study of medical culture and practice; and the development of an online activity guide that other premedical students may adapt to their shadowing circumstances.

**Implementation:** Students reported that they were exposed to new disciplinary perspectives and interprofessional environments that they would not traditionally encounter. Students’ contributions to the shadowing guide encouraged active learning and reflection on the dynamics of effective patient-provider relationships and shadowing experiences.

**Future Directions:** Locally, the class may be scaled for a larger group of premedical students and incorporated into a formal pathway program for premedical students; the content will also be integrated into the clinical medicine course for first-year medical students. Online, the guide will be promoted for use by other institutions and by individuals planning extracurricular shadowing experiences; feedback will be solicited. Tools for evaluating the short- and long-term impact of the course and guide will be developed and validated. Observational and experimental studies of the course’s impact should be conducted.

**Abbreviations:** ICM: Introduction to Clinical Medicine; SCE: Selective Clinical Experiences

## Background

The practice of shadowing, in which students observe healthcare providers, has become a *de facto* requirement for admission to medical school [1]. Exposure to different medical settings during the premedical and undergraduate medical school years theoretically helps students decide whether medicine is a suitable profession for them and influences their choice of specialty [2–4].

Yet recent trends in premedical education suggest that the traditional approach to shadowing is due for an update. The current practice of shadowing has shortcomings. Although formal shadowing programs are intended to increase diversity in the medical profession by helping students with disadvantaged backgrounds gain exposure to the medical field, students who have academic or family connections are still more likely to access shadowing experiences, and to possess the cultural capital to know how to make the most of these experiences [1,2]. Additionally, regardless of student status, the nature, duration, and quality of shadowing experiences varies greatly across providers and institutions [5]. Most existing shadowing guidelines focus on professional standards and ethical compliance rather than pedagogical methods [6]. Educational direction is unspecific: students are simply advised to arrive at their shadowing site and take notes. While medical school admissions committees value applicants’ ability to analyze their shadowing experiences, students often have a limited theoretical or historical framework in which to contextualize their clinical observations, minimal direction about what to observe, and few opportunities to individually and collectively reflect on their observations.

By focusing on establishing the ‘suitability’ of medical school candidates and encouraging prospective medical students to specialize at an early career stage, the traditional approach to shadowing misses an opportunity to foster a richer and broader educational experience. Furthermore, since shadowing is fundamentally an exercise in human observation, the practice holds potential for hands-on instruction in the social dimensions of medicine, which have generated increasing interest among educators and students with the launch of the behavioral science section of the Medical College Admissions Test (MCAT) in 2015.

In response to these needs, a new shadowing course and accompanying online guide (available at http://www.shadowing.healthcare/) are being developed. These resources engage students in exploring facets of medical culture so they may make more fully informed decisions about their specialty in the future. In addition to developing a course at our university, we hope the modular design of the online shadowing guide will be an inspiration for instructors in other institutional contexts and a resource for students who are shadowing outside the purview of a formal program and have had limited guidance about how to structure their experience.

## Development

### Background

The shadowing course Doctoring Undercover was piloted as an upper-level undergraduate elective offered by the medical school faculty through their university’s undergraduate Honors College in the spring of 2016. Enrollment was capped at 20 students. Seventeen students were interested in medicine, two were interested in physical therapy, and one was pre-pharmacy. Students represented a variety of majors, largely drawn from the basic and applied sciences; while there were no pre-requisites for the course, many students had previous extracurricular experience shadowing physicians, both locally and on medical mission trips abroad.

The shadowing sites were determined through collaboration with the directors of the medical school’s required first-year course, Introduction to Clinical Medicine (ICM), which enrolls approximately 135 medical students annually. As a component of ICM, students choose from a list of Selective Clinical Experiences (SCEs) and shadow practitioners at their chosen sites. Although some SCE sites are affiliated with the university’s medical center, sites are chosen based on their ability to expose students to the behavioral, social, and interprofessional aspects of healthcare that are the foci of the ICM course, rather than their proximity to or affiliation with the medical school. Most SCEs are intentionally distinct from traditional shadowing sites such as training programs run by medical school departments or individual physicians’ practices. For example, SCE sites include a hospital-based substance abuse services program that provides motivational interviewing for patients with substance use disorders, a home visitation health education program for at-risk pregnant women, and a multidisciplinary pain clinic.

Eleven SCE directors agreed to extend their programs to include pre-health professions students in Doctoring Undercover; before beginning their site placements, the pre-health students completed the same training and documentation required of undergraduate medical students (i.e., immunization paperwork, HIPAA compliance, and communication with site supervisors about schedules and expectations). Many behavioral science concepts covered in Doctoring Undercover are also scaffolded with the ICM course, along with verbal communication objectives and reflective writing exercises (Table 1). The premedical and medical courses will continue to inform each other as they evolve, supporting the recognized need for scaffolding premedical and medical education in the behavioral and social sciences [7].

**Table 1.** Scaffolding of Doctoring Undercover and Introduction to Clinical Medicine (ICM) courses.

	**Doctoring Undercover**	**Introduction to Clinical Medicine (ICM)**
**Course Organization**		
Student Population	Pre-medical students (sophomores, juniors, and seniors)	First-year undergraduate medical students
Class Size and Format	~ 20 students; seminar style	~ 135 students, divided into small seminar sections of ~ 9 students each
Instructor	Single instructor	Two preceptors for each seminar group (one behavioral science faculty member and one clinical faculty member)
Meeting Times	Two meetings per week; 90 minutes each	1–2 meetings per week; 60–120 minutes each
Duration	One semester	1 year
Clinical Experience Requirement	3–4 shadowing visits to a Selective Clinical Experience site (different sites for students); two introductory observational/interview activities at adjacent health sites (same sites for entire class)	Three visits to a Selective Clinical Experience site; four Longitudinal Clinical Experience visits with a single physician in practice; one visit to the Salvation Army Student Clinic; one patient narrative interview
**Behavioral Science Topics** *Biopsychosocial factors that influence the patient/physician relationship related to:*		
Stress and Wellness	Covered	Covered
Patient Education	Covered	Covered
Pain and Placebos	Not covered	Covered
Grief and Loss	Covered	Covered
Nutrition and Obesity	Not Covered	Covered
Addiction	Covered	Covered
Health Disparities	Covered	Covered
Clinical Ethics	Covered	Covered
**Communication Objectives**		
Role modeling	**Observe** physician/healthcare role models, with a focus on **analyzing** communication skills in a healthcare environment with real patients.	**Observe and interact** with physician/healthcare role models, with a focus on **practicing and observing** communication skills in a healthcare environment with real patients.
Collaboration	**Collaborate** with students, faculty, and practitioners from other disciplines to **identify and analyze** the components of safe and effective patient care.	**Collaborate** with students, faculty, and practitioners from other disciplines to **deliver** safe and effective patient care.
Interviewing Skills	**Analyze** interviewing skills in relation **to one or more of the following**: taking a sexual history, delivering bad news to a patient, assessing for depression/suicidality, and utilizing behavior change/motivational interviewing strategies.	**Demonstrate** advanced interviewing skills by taking a sexual history, delivering bad news to a patient, assessing for depression/suicidality, **and** utilizing behavior change/motivational interviewing strategies.
**Reflective Writing Exercises**		
Format	Blog posts (~750 words each); course begins with instructor-provided prompts and transitions to student generated prompts as students progress	Portfolio assignments; format varies depending on assignment
Frequency	Weekly (mandatory)	Approximately weekly (students can elect to complete additional portfolio assignments to earn a higher grade)
Assessment	Graded with a rubric; 1–2 paragraphs of individualized analytical and editorial feedback from instructor on each post	Graded for completion; Amount of narrative feedback at preceptor discretion
Dissemination and Recognition	All students select two posts for revision and open access publication on course website	Outstanding reflective writing by one or two students is recognized with the ICM Portfolio Award presented annually at convocation

### Course goals and structure

The primary course goals were for each student to leave the class with a deeper understanding of the historical, social, and interpersonal contexts that shape contemporary healthcare and a contribution to an inquiry-based online shadowing guide for fellow pre-health professions students. A secondary goal was to use these experiences to enhance students’ applications to medical school. To achieve these goals, the course was structured in three units. The three units ‘updated’ the medical education maxim ‘See One, Do One, Teach One,’ an aphorism describing the process of moving from observation to mastery of clinical skills, by applying this approach to the social and behavioral dimensions of medicine and using new technologies to facilitate student engagement and reflection.

The first unit, ‘See One,’ was a survey of the academic literature – sociological, historical, anthropological, and bioethical – about the culture and practice of Western medicine. In this unit, students acquired a common vocabulary that enabled them to discuss how today’s medical system developed and how its culture has been studied. For example, in one class session covering ‘insider’ and ‘outsider’ (or ‘-emic’ and ‘-etic’) perspectives on the study of medical culture, students read anthropologist Horace Miner’s classic satire of Western medical practices, ‘Body Ritual Among the Nacirema’ [8]. In groups, they prepared similar satires that denaturalized a taken-for-granted artifact of contemporary medical culture and presented them to the class. Through this exercise, the students adopted an ‘outsider’ perspective and helped one another view familiar medical objects, such as Electronic Health Records and stethoscopes, with fresh eyes. Each week during this unit, students wrote blog post reflections integrating the assigned readings with their experiences and career goals. Throughout the course, the instructor provided individualized analytical and editorial feedback on each post by email.

The second unit, ‘Do One,’ involved observations at selected clinical sites. Before entering the field, students received a site placement by lottery and in-class training in anthropological observational techniques. For example, in one preparatory training activity, students were taught how to map the way the built environment influenced human interaction and health behaviors. In pairs, they selected an on-campus location and took field notes recording their observations. The presentations of their observations revealed a variety of insights about familiar settings. For example, students noted that campus food court design, the timing of meals, and meal plan requirements encouraged the purchase of sodas and ice cream or frozen yogurt over healthier options. Observational techniques, such as mapping, that were acquired during in-class practice activities were then carried out each week in the students’ clinical placements. To guide the clinical observations that took place during this unit, students were also given a weekly observational activity and reflective writing prompt (See Table 2 for instructor-provided activity topics). They produced written blog post reflections and oral presentations of their shadowing experiences. In-class discussions of shadowing experiences enabled students to compare the organizational cultures of various medical sites, the interpersonal interactions of diverse practitioners, and the treatment of different patient populations.

**Table 2.** Instructor-provided and student-generated observational activities for Doctoring Undercover (as of Spring 2016)^a^.

**Instructor-Provided Activities**
Interview a patient’s family member in the hospital cafeteria
Take the bus to a health appointment. Reflect on transportation as a determinant of health
Map and analyze the waiting area at your shadowing site
Analyze health literature (e.g., posters, pamphlets) at your shadowing site
Observe an interaction between a patient and provider. Quantify one aspect of the interaction (e.g., number of interruptions, questions, physical touch)
**Student-Generated Activities**
Navigate the hospital in search of target locations. Observe the architecture and design elements of hospital locations.
Shadow a nurse. Analyze nurse-physician interactions and patient interactions with both professionals.
Shadow at the hospital information station. Observe the different patient populations. Observe the interaction between patients and the medical environment from a non-medical perspective.
Observe interactions between patients’ families and hospital employees that do not provide healthcare (e.g., cafeteria and security workers). Analyze how non-healthcare professionals contribute to the hospital’s ‘positive practice environment’ (PPE).
Explore the process behind scheduling a doctor’s appointment from the point of view of the receptionist, the patient, and the doctor.
Observe how the level of formality in professional dress differs across different health fields and medical specialties. Analyze how the formality of practitioners’ clothing affects patient-provider interaction.
Shadow a chaplain or other spiritual leader. Observe the interactions between the chaplain and a patient or patient’s family member.
Observe a physician breaking bad news to a patient and his or her family member. (This bad news doesn’t have to be a fatal diagnosis; it could be as small as an unpaid bill or a diagnosis of the flu.)
Examine an interprofessional interaction between a doctor, nurse, resident, fellow, or office staff member. Pay attention to facial expressions, body language, tone of voice, and length of conversation.
Navigate and experience a health environment with a visual impairment. Simulate a visual impairment, navigate through the healthcare setting of your choice and take note of the infrastructure, accessibility, and resources available.

^a^The web-based shadowing activity guide will be regularly updated based on new student contributions and community feedback.

The most recent version of the guide, including complete activity descriptions and directions, recommended readings, and prompts for reflective writing, can be accessed at http://www.shadowing.healthcare/guide/

During the third unit, ‘Teach One,’ students formed pairs and, drawing on their experiences at their shadowing sites, composed contributions for the online shadowing activity guide. Each pair created a unique observational activity for the guide, complete with guiding questions for reflection, academic preparatory reading, and a relevant illustration. For example, one pair of students observed that a resident’s clothing choices negatively influenced the ways in which patients and colleagues viewed the authority of the resident’s medical advice. They used this observation to structure a shadowing guide activity in which students analyze the influence of providers’ dress codes on provider-patient interactions (See Table 2 for a list of student-generated activity topics). In preparing their contributions to the guide, students presented drafts of their activities to their classmates for feedback. Based upon their experiences with the assigned observational activities, the students collectively developed a rubric to evaluate their classmates’ contributions to the shadowing guide. The assessment of guide activities was student-led; the instructor averaged the classmates’ ratings and communicated the scores to the pairs of student authors, along with a synthesis of peer feedback and suggestions for revision. This iterative process prompted reflection on the specific elements that constitute a meaningful shadowing experience.

At the conclusion of the class, students refined their contributions to the guide, selected and revised their two best blog posts for final online publication, and brainstormed methods for disseminating their work in the course.

## Implementation

The initial implementation of the pilot course appeared promising. Written qualitative responses were gathered from all 20 students (100% response rate) in answer to two open-ended questions. These responses were gathered separately from the standard satisfaction survey that the university asks students to complete upon the conclusion of each course. In response to the short-answer questions, students described 1) what they learned from the course, and 2) what advice they would give to students in future course iterations. The short-answer responses were thematically analyzed [9]. The illustrative quotes (Table 3) are representative of responses in the sample. No responses were negative in content or tone, and all 20 students indicated the course transformed their perspectives on the medical profession and their understanding of the importance of active learning in medical education [10].

**Table 3.** Thematic analysis of qualitative course feedback.

What did you learn from the course?				
Transformative learning experience	Before the course	Sample quotes	After the course	Sample quotes
1) New perspectives regarding medical science disciplines and allied health fields	*Medicine is a hard science*	‘I hadn’t given much thought to the ’social‘ side of medicine. As a pre-med student, most of my classes can get a bit ''stuck'' in the hard sciences (chemistry, bio-physics) and, frequently, seem a bit monotonous and dull.’	*Medicine is also a social science*	‘This course was some new fresh activity every week. It was a great escape, weekly, from the typical pre-med iron-clad schedule, and I learned more about the ''people'' side of medicine (and myself).’
	*Medicine is a specialized practice*	‘Many aspects of medicine were not apparent.’	*Medicine is a multi-disciplinary enterprise*	‘The class used us to get deeply involved with shadowing sites that were non-traditional. From these sites, we got to see low SES [patient populations], or spirituality’s role in health, or drug abuse management. All these critical sites help develop our perspective of medicine.’
2) New techniques for improving the patient-provider relationship	*Focus on acquisition of academic knowledge*	‘I was excited to get my grades up and go to med school to learn how to do everything I’ll need to know to treat my patient.’	*Awareness of the importance of the patient-provider relationship and familial context*	‘There is more to being a doctor than I used to think. I want to have good doctor-patient relationships, and we talked how to improve this relationship. When we talked to a family member of a patient in the hospital it opened my eyes and reminded me to also pay attention to [patients’ families].’
	*Focus on clinical procedures*	‘When I used to shadow, the only aspect I paid attention to was the actual procedure.’	*Focus on techniques for improving patient-provider communication*	‘I feel as though I have learned many tips about what to do and what not to do by observing and will certainly remember how important good bedside manner and empathy are.’
3) Increased awareness of psychological, social and cultural aspects of medicine	*Little or no exposure to social determinants of health*	‘I had little to no actual knowledge on the social determinants of health and how these [affect medicine.]’	*Awareness of the physician’s position in a larger system of social and policy determinants*	‘By being educated on some different medical policy issues, I gained an awareness of my own stance on these issues.’
	*Belief that access to medical care was the sole determinant of health.*	‘Before the class, most of my ideas about the shortcomings of the healthcare system were based on the lack of access of many to healthcare.’	*Awareness that healthcare access is only one determinant of health*	‘I developed an understanding of the interplay between social conditions and healthcare treatment access. I will always remember [the activity in which we used] public transportation to attempt to get to an appointment on time or witnessing a caring nurse consistently support a man with severe Alzheimer’s. Knowledge of treatments and physiology is not enough. We must meet our population where they are. ’
4) Acquisition of strategies for personal reflection on medical practice	*Shadowing is a passive activity*	‘[I thought shadowing was] simply watching a professional and getting an idea of what a typical day is like.’	*Shadowing is a form of active learning*	‘[I learned that] you can also gain essential knowledge and experience to make you a successful doctor/nurse/etc. as long as you actively look for it. Shadowing is not supposed to be passive!’
	*Limited experience with reflective writing*	‘I have only written scientific papers. I had never written anything like a blog post before.’	*Reflective writing leads to deeper personal understanding*	‘I learned that writing about a topic is the best way to figure what I think about that topic. Writing personal blog post entries rather than formal essay pieces was honestly refreshing. I felt like I could truly write my thoughts in my own voice.’

What advice would you give to future students in the course?				
Transformation in learning approach	Before the course	Sample quotes	After the course	Sample quotes
1) Be open-minded and go ‘out of your comfort zone’	*Expectations about which shadowing sites would be most beneficial*	‘I was disappointed with my shadowing site at first, but have had one of the most rewarding experiences to date!’	*Student empowerment in shaping the learning experience*	‘Have an open mind. You will get as much out of this course as you’re willing to put into it. Be creative. There is no opinion or idea that is wrong. Question yourself. Allow this class to help you grow.’
	*Anxiety about asking questions or speaking in class*	‘Don’t be afraid to ask questions or talk to people, whether patients, classmates, or doctors.’; ‘Don’t be like me and stress out about talking to the class.’	*Understanding of the role of discomfort in the learning process*	‘In this course especially you will gain as a direct result of what you invest. When participating in an activity, go out of your ’comfort zone‘ and your understanding of course content will become personally meaningful.’
2) Put effort into course reflections (field notes, blog posts, shadowing activities)	*Skepticism about the value of student-directed course activities*	‘At first I was very skeptical about writing blog posts and creating a website.’	*Recognition of personal and professional value of student-directed activities*	‘Put a lot of effort into the [blog posts and shadowing guide]. These activities have shed a lot of light on what my personality is as well as given me great experiences as I pursue a medical degree.’
	*Belief that course activities other than direct shadowing experience were superfluous*	‘Don’t rush through the [other parts] of the course because you are only interested in the hands-on shadowing. There is a great deal to be learned that will play a role in what you get out of your shadowing experiences.’	*Belief that assignments facilitate lifelong professional identity formation*	‘Taking really good field notes will help you in reflecting in years to come.’

In keeping with the exploratory nature of a pilot qualitative assessment [11], the overarching themes of transformative and active learning emerged from the data and were not determined *a priori*. For example, in describing what they learned in the course, students might have listed new terminology or practical skills. Instead, all students described changes in perspective consonant with the educational concept of learning as a transformative process that challenges and alters students’ self-understanding, beliefs, and/or behaviors [10]. The transformative learning experience described in students’ feedback expressed four sub-themes: 1) new perspectives regarding medical science disciplines and allied health fields, 2) new techniques for improving the patient-provider relationship, 3) increased awareness of the psychological, social, and cultural aspects of medicine, and 4) acquisition of strategies for personal reflection on medical practice.

In their coursework, the students analyzed shadowing experiences through a social science lens that goes beyond narrow observations of symptom identification and doctor-patient interaction, and places these interactions in wider social, spatial and temporal contexts. Analyzing how macro-level factors influence clinical encounters and evaluating observational activities for the online guide encouraged students’ reflection on the dynamics of an effective shadowing experience. Responses to the second question suggest that students internalized an active, self-directed approach to medical education, in which students take responsibility for shaping their learning experience rather than memorizing content in preparation for standardized tests. The types of advice respondents offered to future students expressed two themes: 1) be open-minded and go ‘out of your comfort zone’, and 2) put effort into course reflections. For some students, cultivating an active learning approach made the quality of the shadowing experiences less dependent upon the personality and specialty of site supervisors: ‘I have learned that no matter what setting you are shadowing in, there is always some valuable experience that you can take away from it and even apply to life outside the classroom,’ wrote one respondent (See Table 3 for themes and illustrative quotes).

In addition to preparing students to actively participate in future clinical training [12], it is hoped that the course will enhance students’ medical school applications. The program has been brought to the attention of the university’s medical school admissions directors, and several class sessions were devoted to assisting students with applying course experiences to their application materials. For example, students received coaching in how to discuss their shadowing experiences in mock admissions interviews, as well as guidance in incorporating elements from course blog posts into personal statements. An in-class panel of admissions committee members and officers answered student questions about the admissions process. Five students included course material in applications for the medical school admissions cycle that began immediately following the course’s conclusion. If growing cohorts of Doctoring Undercover alumni successfully apply and matriculate to the university’s medical school, increased institutional awareness of the unique preparatory experience provided by the course will be an additional benefit of the new shadowing program. Local institutional change is an essential initial step in modernizing the traditional approach to shadowing.

## Future directions

Multiple levels of course development are planned. Locally, the class may be expanded to a larger group of premedical undergraduates at the university, perhaps by offering the course more frequently or simultaneously offering multiple course sections. The course may also serve as an experiential learning component of a more comprehensive premedical curriculum in Medical Behavioral Science, perhaps in the context of a pathway program developed in partnership with medical school faculty and admissions officers, premedical advisors, and the university’s undergraduate administrators and educators. The proposed pathway program would supplement the standard premedical curriculum with courses focused in Medical Behavioral Science and provide pathway students with tailored research mentorship in the social sciences and additional academic advising regarding medical school admissions. The addition of new shadowing sites is planned. Some Doctoring Undercover course content will also be adapted and integrated into the ICM course for medical students.

The online shadowing guide also has the potential to serve as a resource for instructors and students in other educational contexts. We are developing animated instructional videos for the guide that will cover the general shadowing procedures currently addressed in face-to-face class meetings. The videos will address logistical topics such as preparing to shadow; professional behavioral standards for shadowing; and tips for incorporating shadowing experiences into medical school application materials.

Along with the videos, the guide activities could be incorporated into courses at other institutions or into extracurricular shadowing programs. The guide will be disseminated using both traditional methods (academic presentations and publications) and new media methods (social media, blogs, list-servs) of scholarly communication. Surveys for instructor and student users of the guide will be developed, and feedback will be solicited by email and through a link on the guide website. The guide will be periodically updated to reflect new student contributions and responses to user feedback. Tools for assessing the short- and long-term impact of both the course and guide on student perspectives, medical school admissions, and overall career trajectories may be developed.

Formal study of the effectiveness of the course material will be essential to its future adoption and impact. The hypothesis that the course material promotes a transformative and active learning experience could be explored through the delivery of pre- and post-course assessments related to these constructs. It is hoped that research will be undertaken once the curriculum has reached an appropriate stage of development and the students impacted constitute an appropriate sample size for quantitative study. Future studies may include, for example, comparing the attitudes and outcomes of Doctoring Undercover students with those of students who engaged in traditional shadowing experiences, or comparing the attitudes and outcomes of students who were randomly admitted to the course versus those who were not. The development and validation of an assessment tool specifically related to premedical student shadowing would be valuable for the further assessment of this course material and for the medical education community at large.

Ideally, the dissemination and further evaluation of this course material to students, pre-health professions counselors, shadowing supervisors, and medical educators will influence changes in the culture and practice of shadowing itself.

The course may influence change by educating students about the behavioral and social dimensions of medicine in an experiential, rather than didactic, manner; by expanding the ways in which premedical advisors and medical school admissions committee members view the purpose of shadowing; by supplying activities that encourage student engagement and foster more rewarding mentoring experiences for shadowing supervisors; and by offering an adaptable framework for structuring an educational experience whose outcomes have long been subject to personal chemistry and circumstance.
